# Pathological post-obstructive diuresis following obstructive uropathy due to constipation in the post-partum period: a case report

**DOI:** 10.1186/s12884-021-04218-1

**Published:** 2021-11-01

**Authors:** Mohamed Rishard, Thalagala Kossinnage Maheeka Kossinna, Mohamed Nazar Latiff, Susantha De Silva, Mohamed Hilmy Mohamed Asmath

**Affiliations:** 1 Department of obstetrics and gynecology, Faculty of Medicine, University of Colombo, Colombo, Sri Lanka; 2 Nawaloka Hospitals PLC, Colombo, Sri Lanka; 3 National Hospital of Sri Lanka, Colombo, Sri Lanka; 4 Department of Urology, National Hospital of Sri Lanka, Colombo, Sri Lanka; 5 Colombo South Teaching Hospital, Kalubowila, Sri Lanka

**Keywords:** Constipation, Post-obstructive diuresis, Obstructive uropathy, Post-partum morbidity, Fecal impaction

## Abstract

**Background:**

Constipation during pregnancy is not uncommon. Usually, this does not warrant extensive evaluation and settles with minor interventions or lifestyle modifications. Severe fecal impaction in chronically constipated patients can rarely lead to obstructive uropathy. Relief of obstruction can result in a diuretic phase which may be self-limiting or pathological. However, occurrence of pathological post-obstructive diuresis as a result of severe constipation is an extremely rare complication during pregnancy and puerperium which can even be fatal if not promptly diagnosed and adequately monitored and timely intervened. We describe the management of a pathological post-obstructive diuresis which occurred in the immediate postpartum period after treatment of severe constipation and obstructive uropathy.

**Case presentation:**

A woman who had undergone an emergency caesarean section due to deep transverse arrest 1 week ago, presented with fecal impaction and anuria. On relief of urinary obstruction which had developed secondary to fecal impaction, she developed pathological post-obstructive diuresis. Careful and timely monitoring with exact fluid replacement, correction of electrolyte imbalances and multidisciplinary care ensured complete recovery of the patient.

**Conclusions:**

Despite obstructive uropathy being uncommon in obstetric practice, clinicians need to have a high index of suspicion to monitor and promptly manage the potentially life-threatening condition of post-obstructive diuresis in pregnant and puerperal women undergoing urinary tract decompression. Due to unreliability of laboratory cutoff values in pregnancy and puerperium, a more vigilant and multidisciplinary approach with lower threshold for intervention is more prudent in the management of these patients.

## Background

The terms postnatal period and postpartum period are interchangeably used to refer to the first 6 weeks (42 days) following childbirth and is further subdivided as immediate (first 24 h), early (day 2 – day 7) and late (day 8 – day 42) phases of the postpartum period [1]. During this time, physiological changes of pregnancy are slowly reverting to the pre-pregnancy state. This period is riddled with a myriad of maternal illnesses. While most of these conditions may present during the first 1–2 weeks, among the scores of postpartum morbidities such as postpartum infection, anemia, perineal tears, urinary tract infection, and depression, are numerous and complex pathologies with onset, severity and duration of illness varying vastly [2].

Early detection and adequate and timely management are crucial in these conditions to reduce the risk of developing acute life-threatening conditions that may fall into obstetric near-miss category [3] as well as serious long term sequalae which may lead to lifelong co-morbidities.

Constipation during the postpartum period is not an uncommon disorder. It is defined as low frequency and difficulty in passing stools which can be diagnosed following the criteria of less than 3 bowel movements a week, hard stools and/or difficulty in passing stools. Causing symptoms such as pain or discomfort on defecation, straining, hard lumpy stools, and a sense of incomplete bowel evacuation, constipation can be severe and may affect the quality of life of the woman significantly while also diverging her attention from the newborn who needs constant care [4].

While obstructive uropathy has been described as a complication of chronic constipation across literature [5–7], it is rare to find constipation leading to obstructive uropathy outside of the extremes of age. However, constipation following delivery, leading to obstructive uropathy and severe post obstructive diuresis during the postpartum period is an extremely rare set of sequalae. It’s rarity if such that there are no cases of development of pathological POD upon relieving the constipation during pregnancy or in the postpartum period reported in the literature. This unreported consequence of constipation in pregnancy warrants the attention of the obstetrician not only to enlighten him/her regarding this interesting and complex phenomenon in the context of pregnancy but also to warn him/her to have a high degree of suspicion to diagnose and to take careful measures and precautions when dealing with such clinical situations such as lowering threshold for intervention considering the altered physiology of post-partum period and early involvement of the nephrology team.

To this end, we describe a clinical case that presented with obstructive uropathy caused by severe constipation developing into severe post-obstructive diuresis.

## Case presentation

A 39-year-old woman in her 3rd pregnancy booked with us at 12 weeks of gestational age. Her previous pregnancies had culminated in normal vaginal deliveries. She is a housewife with a moderately active lifestyle. Aside from a history of on and off constipation, her past medical history was unremarkable and her current pregnancy was managed as a low-risk pregnancy. All her booking visit investigations including thyroid status were normal. She was on calcium supplements throughout the pregnancy and iron supplementation in second and third trimesters. Her booking BMI was 28 and total weight gain was 9 kg.

At 39 weeks of gestation, she went into labour spontaneously. Following approximately 4 h of the latent period, she had a spontaneous rupture of membranes. On admission to the labour ward, her cervical dilatation was 4 cm and well effaced with one mild contraction per 10 min. No rectal enema was given on admission to labour ward. Second review after 4 h revealed the similar vaginal examination findings while she was experiencing only 2 moderate contractions per 10 min. After careful evaluation, oxytocin infusion (5 mIU/ml) was commenced and maintained to achieve 3–4 contractions per 10 min. She opted to have nitrous oxide and intramuscular pethidine as pain relief and had liberal oral fluids during active first stage of labour. Active phase of the first stage lasted 5 h in total, after which active pushing in the second stage was attempted for nearly 45 min. At this stage, a moderate caput had developed with grade 2 moulding and the diagnosis of deep transverse arrest was made. The attempt at manually rotating the fetal head in the labour room failed. Since the patient declined a trial of instrumental delivery, an emergency cesarean section was performed. During the cesarean section, the uterine incision was noted to have a right sided extension towards the vagina which was sutured carefully. No bladder damage was noted. Total blood loss was approximated at 400 ml, and her baby weighed 2.9 kg. She was commenced on oral fluids 2 h postoperatively and mobilized after 6 h from surgery. Standard post-operative bladder care, catheter care and fluid management were given. Urinary catheter was removed on post-operative day 1 and the patient was discharged on the second post-operative day after spontaneous bowel opening with paracetamol and NSAIDs for analgesia, oral antibiotics for 5 days with prophylactic dose of low molecular weight heparin for 7 days and iron supplements for 6 months.

One week later she had presented to the general practitioner with a history of no bowel movements for 6 consecutive days and anuria for the last 3 days. She was prescribed stool softeners and a rectal enema and was directed to the emergency department by the general practitioner. However, patient did not present to the emergency department initially as she had a bowel movement. Over the next day she complained of severe abdominal discomfort and progressive abdominal distention with difficulty in breathing and persistent anuria prompting her to ultimately present to emergency treatment unit.

On admission she was hemodynamically stable, and her abdomen was distended with evidence of free fluid. As she had undergone a second stage cesarean section, the team decided to catheterize and rule out the possibility of a bladder injury causing urine leakage into the peritoneal cavity. Catheter drained 1 l of clear urine and subsequent ultrasound of the abdomen and pelvis showed normal bladder contour, normal ureters and moderate amount of peritoneal free fluid. Her renal function tests showed evidence of acute kidney injury, which were as follows:

eGFR − 16.67 ml/min/1.73m^2^.

Blood Urea − 150.5 mg/dl.

Serum creatinine − 3.32 mg/dl.

Digital rectal examination revealed significant fecal loading and manual dis-impaction was done; followed by rectal enema. Subsequently she was commenced on oral laxatives. Upon further evaluation of the patient’s history, it was revealed that she had constipation throughout the latter part of her pregnancy which she had failed to mention in previous appointments with her caregivers.

Over the next 12 h the patient produced 12 l of urine. Her laboratory investigations revealed hyponatremia with serum sodium level of 123 mmol/l (reference range – 136- 145 mmol/l). Patient’s urine protein level was 8.1 mg/dl (reference range – 0 – 15.0 mg/dl) and urine protein: creatinine ratio was 0.38 (reference range- less than 0.2). The patient had a serum osmolality of 301 mOsm/kg (reference range – 275- 295 mOsm/kg) with a urine osmolality of 270 mOsm/kg (reference range – 700-1500 mOsm/kg) showing an inability to concentrate urine despite the physiological response of serum hyperosmolality. Her urine full report and urine culture were unremarkable with urine specific gravity at 1.015. Spot urine sodium was 66 mmol/l.

Her other laboratory investigations were as follows: serum potassium – 5.1 mmol/l (reference range – 3.5 – 5.1 mmol/l), serum chloride – 89 mmol/l (reference range – 98 – 107 mmol/l), serum calcium – 8.2 mg/dl (reference range 8.6–10.0 mg/dl), serum magnesium – 1.4 mg/dl (reference range 1.6–2.6 mg/dl). Her liver function tests were normal and 2D echocardiogram had no abnormal findings.

Upon advice from the consultant nephrologist, fluid replacement was done with liberal oral fluids and intravenous fluid replacement at less than 10% of the previous hour urine output. Oral calcium supplements, oral sodium chloride replacement and single dose of intravenous magnesium sulphate infusion of 1 g was added to her treatment regime.

Marked clinical improvement of the patient was seen over the course of the initial 12 h with resolution of her abdominal discomfort and difficulty in breathing. Subsequent 24 h saw persistent polyuria with the patient’s condition being stable. Close monitoring of the urine output hourly ensured proper fluid balance with serial laboratory values for serum electrolytes, urine and serum osmolality and renal function assessment every 12 h fortifying the ongoing management. Over 36 h after onset of diuresis patient’s renal function and other laboratory values returned to normal with the settling of polyuria more than 48 h after onset.

The patient was discharged on the day after resolution of polyuria with a foley catheter in-situ which was subsequently removed a week later. Patient resumed normal voiding of urine after catheter removal. Post-void residual volume 1 week after catheter removal was ultrasonically confirmed to be less than 50 ml.

## Discussion

Pregnancy is a physiological phenomenon characterized by numerous multisystemic anatomical and physiological changes. Such gastrointestinal changes in pregnancy contribute to constipation, seen in as many as 10–40% of pregnant women [8]. In our patient, pregnancy associated hormonal and anatomical changes, dietary habits and a more sedentary lifestyle during pregnancy and puerperium would have all contributed to worsening her pre-existing constipation [4] leading to fecal impaction.

We believe that undiagnosed constipation may have contributed to the deep transverse arrest of labour that resulted in the patient undergoing caesarean section [9]. Cochrane review concludes that routine treatment with enema in labour should be discouraged [10]. However, that should not preclude us from careful history taking and mindful evaluation that could prompt caregivers to intervene if significant constipation is identified.

Post-operative period in this patient would have exacerbated the constipation she experienced in pregnancy leading to fecal impaction, compounded by poor fluid intake, inadequate mobilization and medication such as opioid analgesics and iron supplements [11, 12].

This patient presented with obstructive uropathy complicated by peritoneal free fluid. Exact mechanism of peritoneal free fluid accumulation in this patient is unclear but can be a complex pathophysiology including acute renal failure [13] and pregnancy related lymphatic drainage obstruction [14] all would have played a role.

Fecal impaction causing obstructive uropathy is described in literature in elderly and in paediatric age groups [5–7]. In adult females, pelvic tumors, gravid uterus, gynaecological malignancies and uterovaginal prolapse are several aetiological factors for obstructive uropathy [15]. However, there are no documented cases of fecal impaction leading to obstructive uropathy and consequently post-obstructive diuresis, in the post-partum period.

With the relief of the obstruction as many as 0.5–52% of patients may experience post-obstructive diuresis which can be potentially life threatening [16]. Post-obstructive diuresis can be defined as urine output of more than 200 ml/hour over more than 2 consecutive hours immediately following relief of the obstruction or a urine output of more than 3000 ml/24 h [17].

This is a normal physiological response adopted by the organism to remove the accumulated excess of water and solutes during the period of urinary obstruction. However, in some patients, this physiological response is prolonged past the stage of normal solute concentrations and normovolemia causing extended diuresis leading to water and electrolyte abnormalities such as severe dehydration, hyper/hyponatremia, hypokalemia, hypomagnesemia, metabolic acidosis and at the most extreme, hypovolemic shock and even death if not duly managed [16–18]. The initial physiological post-obstructive diuresis is self-limiting and usually resolves by 24 h, whereas pathological post-obstructive diuresis lasts longer than 48 h with associated water and electrolyte imbalances, needing careful monitoring and appropriate fluid and electrolyte management [17].

Pathophysiology of post-obstructive diuresis is complex and can be discussed under several processes which includes, reduction of the medullary concentration gradient caused by vascular washout and downregulation of sodium transporters in thick ascending limb of loop of Henle, ischemia and loss of juxtamedullary nephrons preceded by reduced glomerular filtration rate, and nephrogenic diabetes insipidus caused by reduced response to antidiuretic hormone by the collecting duct [16–18].

These mechanisms lead to a mixed water-solute type diuresis where urine osmolality is 100-300 mmol/kg as was seen in this patient [19]. Spot urine sodium level of more than 40 mEq/l is suggestive of renal tubular injury which, in turn, points to a more likely scenario of pathological post-obstructive diuresis [16]. The duration of polyuria which lasted more than 48 h after relief of obstruction in this patient, is also suggestive that a pathological post-obstructive diuresis occurred while the timely management with the involvement of the nephrologist and the obstetric team stabilized patient’s condition.

Volume of urine immediately drained following relief of obstruction being more than 1500 ml may indicate that the patient would be developing post-obstructive diuresis. However, there is no clear evidence on the factors which may indicate the physiological post-obstructive diuresis developing into pathological post-obstructive diuresis. Some studies, however, point towards pre-existing renal damage, congestive heart failure and volume overload as well as dizziness and central nervous system depression to be predictors of the above development, none of which this patient exhibited [16, 17]. Therefore, a high index of suspicion needs to be exercised following relief of urinary tract obstruction regardless of the demographical and biological risk factors to timely intervene in post-obstructive diuresis, even in the obstetric practice.

Pregnancy brings about multiple changes to the renal system. Glomerular filtration rate increases up to 50% and renal blood flow increases up to 80% compared to the non-pregnant values, driven by volume expansion and vasodilatation. Water and electrolyte hemostasis by the renal tubules are altered resulting in mildly increased proteinuria, glucosuria, lower serum osmolality and lower serum sodium concentration. Mechanical obstruction of the ureters leading to hydronephrosis is also commonly seen in pregnancy. Coupled with anatomical changes of the pelvicalyceal system, these anatomical and physiological changes can affect the clinical picture and laboratory values when in pregnancy [20].

While the patient is still recovering from the pregnancy, post-partum period is also a state of altered physiology albeit being an evolving state of returning to non-pregnant state. Therefore, laboratory investigations and clinical symptoms and signs may vary from the cutoff values given for the non-pregnant female population. While studies are being undertaken to quantify and determine the cutoff laboratory values for pregnancy and postpartum, no such parameters are yet established for multiple investigations we need to consider in assessing a patient such as this [21, 22]. Due to this unreliable nature of the diagnostic tools during the puerperium, obstetric team needs to have a high degree of suspicion and lower threshold for intervention for post-obstructive diuresis.

## Conclusions

Constipation is aggravated by hormonal, anatomical and lifestyle practices in pregnancy and puerperium, which can lead to debilitating sequalae. Although not essential or recommended to medically manage constipation in pregnancy and puerperium, intervention maybe necessary for more severe cases, which is to be decided on a case-by-case basis. Constipation leading to obstructive uropathy with resultant post-obstructive diuresis is not well documented in the pregnancy or puerperium. However, it is crucial to have a high index of suspicion, in a patient with postpartum obstructive uropathy for timely intervention and involvement of a multidisciplinary team in case of developing post-obstructive diuresis. Since the physiology is altered in pregnancy and has yet to return to normal adult physiology in the puerperium, it is essential to account for the altered standards for laboratory values and clinical evaluation. Therefore, postpartum women undergoing this disease progression may need earlier intervention than in other adults, which can further make the waters murkier for the diagnosis and management of these patients.

## Data Availability

All data generated or analyzed during this study are included in this manuscript.

## Abbreviations

BMI: Body Mass Index
NSAIDs: Non-Steroidal Anti-Inflammatory Drugs
eGFR: estimated Glomerular Filtration Rate
